# N7-(carboxymethyl)guanine-Lithium Crystalline Complex: A Bioinspired Solid Electrolyte

**DOI:** 10.1038/srep24499

**Published:** 2016-04-19

**Authors:** Dipak Dutta, N. Nagapradeep, Haijin Zhu, Maria Forsyth, Sandeep Verma, Aninda J. Bhattacharyya

**Affiliations:** 1 Solid State and Structural Chemistry Unit, Indian Institute of Science, Bangalore-560012 (Karnataka), India; 2 Department of Chemistry, Indian Institute of Technology, Kanpur, Kanpur-208016 (UP), India; 3 Centre for Nanosciences, Indian Institute of Technology, Kanpur, Kanpur-208016 (UP), India; 4 Centre for Environmental Science and Technology, Indian Institute of Technology, Kanpur, Kanpur-208016 (UP), India; 5 Institute for Frontier Materials, Deakin University, Waurn Ponds, VIC3216, Australia

## Abstract

Electrochemical device with components having direct significance to biological life processes is a potent futuristic strategy for the realization of all-round green and sustainable development. We present here synthesis design, structural analysis and ion transport of a novel solid organic electrolyte (G7Li), a compound reminiscent of ion channels, derived from regioisomeric N7-guanine-carboxylate conjugate and Li-ions. G7Li, with it’s in-built supply of Li^+^-ions, exhibited remarkably high lithium-ion transference number (= 0.75) and tunable room temperature ionic conductivity spanning three decades (≈10^−7^ to 10^−3^ Ω^−1^ cm^−1^) as a function of moisture content. The ionic conductivity show a distinct reversible transition around 80–100 °C, from a dual Li^+^ and H^+^ (<100 °C) to a pure Li^+^ conductor (>100 °C). Systematic studies reveal a transition from water-assisted Li-ion transport to Li hopping-like mechanism involving guanine-Li coordination. While as-synthesized G7Li has potential in humidity sensors, the anhydrous G7Li is attractive for rechargeable batteries.

The severe safety hazards and environmental concerns associated with the usage of liquid electrolytes in electrochemical devices have been the focus of attention in research and development for several decades^1^,^2^,^3^. For rechargeable batteries based on lithium, the major issues with liquid electrolytes are high volatility and flammability combined with high rates of the reactivity with the electrodes^4^. One school of thought to tackle the severities associated with molecular liquids and enhance the safety has been to replace them by solid electrolytes^5^,^6^. In this context, several glassy, inorganic and organic polymer solid electrolytes have been demonstrated as potential candidates for all-solid state electrochemical devices such as batteries, fuel cells, etc^7^,^8^,^9^,^10^,^11^. However, an overwhelming majority of them have not been able to successfully transcend beyond the precincts of laboratory-scale demonstrations^12^,^13^,^14^,^15^,^16^,^17^,^18^ which led to the predominant usage of liquid electrolytes^19^. The other important issue of sustainability, so far has not been vigorously explored. Many of the commercial liquid electrolytes including room temperature ionic liquids are derived from materials with limited abundance and their purest form is hard to achieve^20^. This is of paramount importance for large scale electrochemical applications as they are not only going to increase device cost, but also going to cause larger irrecoverable damage to Earth’s ecology and environment.

Similarities in the chemical composition and structural motifs has triggered an urge for expanding the horizon of organic biomaterials to non-biological applications^21^,^22^,^23^. Recently, the effective use of organic biomolecules like DNA and peptides as structural templates for synthesis of energy storage materials has been demonstrated^24^,^25^. Unlike conventional inorganic materials, organic biomaterials can be obtained from renewable sources and offer higher flexibility in terms of ease of processability and tunability of chemical compositions, which eventually will tremendously aid in improving the efficiency of the device. Guanine, a natural heterocyclic nucleic acid constituent, is a known electronic conductor (conductivity ≈ 10^−15^ Ω^−1^ cm^−1^ at 400 K and under vacuum of 10^−6^ mm of Hg)^26^ due to the hole-hopping mechanism in oligonucleotides^27^,^28^,^29^. Further, addition of metal complexes to nucleic acids is known to aid hole migration, a vital feature in long-range charge transport in such systems^30^. This approach is expected to offer new avenues to construct DNA-based electronic devices and related technologies^31^,^32^,^33^. Additionally, guanine supports formation of extended supramolecular architectures, at times in the presence of small cations^34^,^35^. These supramolecular architectures may sustain long-range ion transport and may be used to design bio-conductive materials^36^ and also hold promise as electrolytes in electrochemical applications. Herein, we report for the first time a guanine based solid organic crystalline electrolyte G7Li, which has crystalline structure reminiscent of ion channels and having direct significance to biological life processes. G7Li is synthesized from N7-(carboxymethyl)guanine with Li ions and its ion transport properties are correlated to its crystal structure via extensive studies involving ionic conductivity and static ^7^Li solid-state NMR spectroscopy (^7^Li line-shapes and motional narrowing). Notably, G7Li exhibited tunable room temperature ionic conductivity spanning over three orders in magnitude as a function of humidity, thus providing a novel readily-accessible bio-friendly electrolyte from guanine (an essential purine nucleobase).

## Results and Discussion

**Figure 1:**
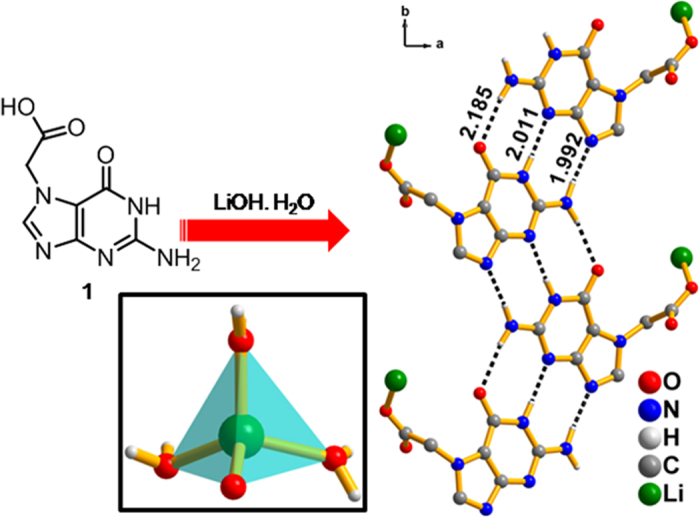
Synthesis of G7Li from 1. (right) guanine-guanine hydrogen bond interactions in G7Li. Inset: distorted tetrahedral geometry of Li ion in G7Li.

**Figure 2:**
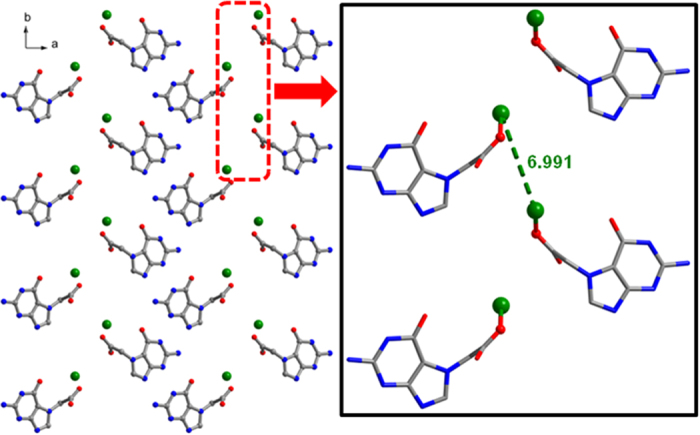
Alignment of Li ions in G7Li, when viewed along the *c*-axis; inset: observed Li−Li distance in G7Li.

Complexation of N7-(carboxymethyl)guanine (**1**) with LiOH·H_2_O, followed by slow evaporation resulted in colorless crystals of G7Li (c/f Tables S1 and S2 in the Supplementary Information, ESI). Single crystal X-ray analysis reveals that G7Li crystallized in monoclinic space group P2_1_/*c* and the asymmetric unit composed of an anionic guanine derivative (**1**), one Li-ion and three water molecules in which **1** and the water molecules are directly bound to the lithium ion revealing a distorted tetrahedral geometry (Li1O1 = 1.933 Å). Notably, modified guanine moieties interacted through the Watson−Crick face and the extended sugar edge, due to the availability of free N9 acceptor site. These interactions are supported by six hydrogen bonds (N2−H2B…N9 = 1.992 Å; N2−H2A…O6 = 2.185 Å; N1−H1…N3 = 2.011 Å) (Fig. 1), resulting in the formation of infinite guanine ribbons, when viewed along the *c*-axis^37^. This DDA…AAD triple hydrogen bonding sequence between guanine moieties is scarce in the literature (where D = donor, A = acceptor)^38^,^39^. These guanine ribbons are further stabilized by π-π interactions involving parallel offset rings and exhibited a ladder-like structure, when viewed along the *b*-axis (c/f Figure S1 in the ESI). Infinite array of lithium ions are observed in the lattice when viewed along the *c*-axis. The lithium ions are equispaced (*d*_Li1−Li1_ = 6.991 Å, Fig. 2) and the distance between them is longer than the sum of the van der Waals radii (3.64 Å) and is obviously longer than the sum of ionic radii (1.86 Å)^40^ as well. The alignment of lithium ions and the distances between them in the crystal lattice (when viewed along different crystallographic directions) is further depicted in Figure S2. Surprisingly, the carbonyl oxygen O^6^ of guanine did not show any direct interaction with lithium ions instead interacted with lithium bound water molecules which in turn connected through carboxylate oxygens (c/f Figures S3 and S4). Thus the G7Li on one hand strongly resembles a ceramic Li-ion conductor to a great extent in terms of physical appearance (in the form of powder with a lower melting temperature). On the other hand, the G7Li also resembles an ionomer due to the built-in Li^+^-ions in the intrinsic structure. These Li-ions are expected to sustain Li^+^-ion transport and no extra addition of Li-salt is required. This is an added advantage over polymer electrolytes where additional Li-salt has to be added to sustain conductivity.

**Figure 3:**
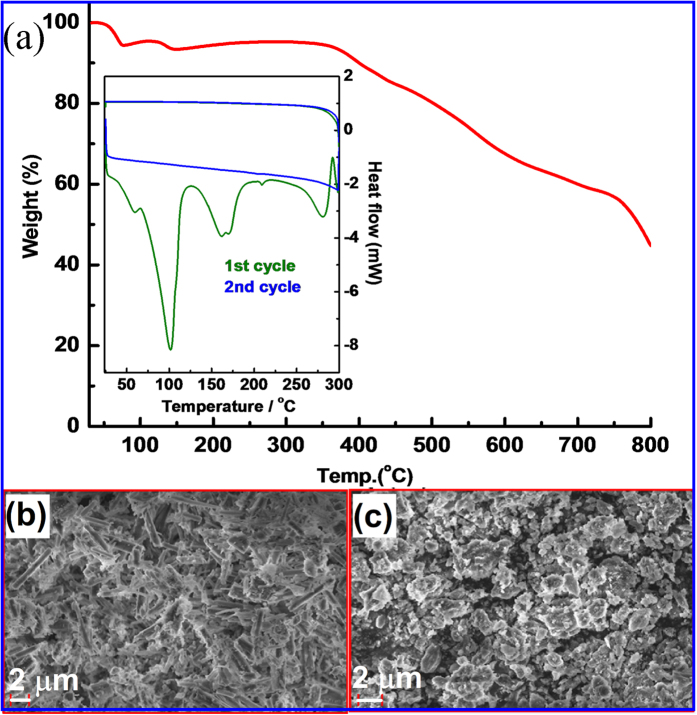
(**a**) TGA profile of G7Li (33–800 °C; heating rate 5 °C/min, N_2_ atmosphere) (inset: DSC profile of G7Li (heating rate 5 °C/min, N_2_ atmosphere)). SEM images of (**b**) as-synthesized G7Li and (**c**) after heating the sample to 300 °C (Scale: 2 μm).

Thermal behaviour of G7Li is studied by thermogravimetric analysis (TGA) which is depicted in Fig. 3a. The initial weight loss of 6.2% (at 80.5 °C) corresponds to the loss of two water molecules from the complex. On further heating, a cusp is observed at around 100 °C, followed by rapid decomposition after 300 °C (stable up to ~375 °C). The thermal (decomposition) properties of G7Li are better than many of the reported promising soft-matter (polymer) electrolytes^41^,^42^,^43^. The thermal stability of G7Li up to ~375 °C, which is sufficient for majority of the electrochemical applications, however, is appreciably lower than any typical ceramic conductor. We envisage that this lower thermal stability range of G7Li is not going to be a major disadvantage as this will be easily offset by its’ higher degree of biocompatibility and sustainability compared to majority of the prevalent electrolytes. Room temperature (RT) powder X-ray diffraction (PXRD) patterns of as-synthesized G7Li and sample pre-heated to 300 °C are in good agreement with the simulated diffraction patterns (c/f Figure S5 in the ESI). Subsequent SEM analysis revealed a rod-like morphology for the as-synthesized G7Li sample which is completely destroyed on heating the sample at 300 °C (c/f Fig. 3b,c). However, the destruction of the rod-like morphology does not imply the loss of crystallinity of the sample. This is confirmed by the PXRD pattern (Figure S5), DSC and ionic conductivity results (vide infra).

**Figure 4:**
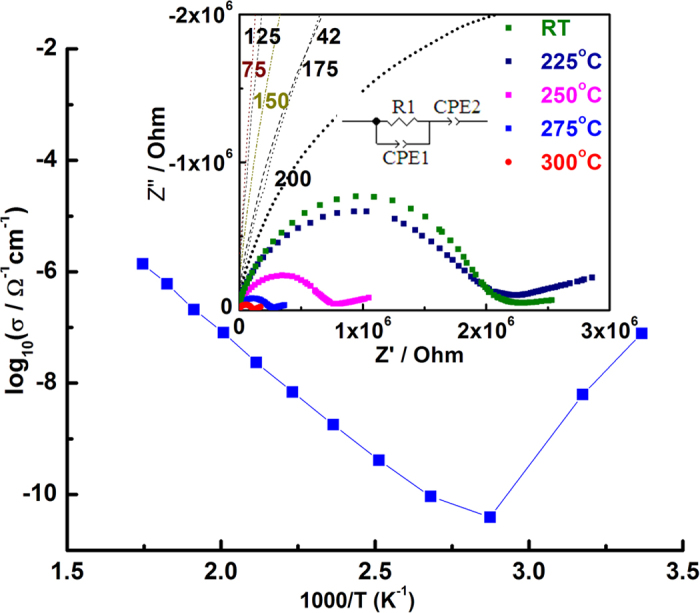
Arrhenius plot of the conductivity versus temperature (25–300 °C) of G7Li at 39% relative humidity (RH) (ambient condition). Inset: Nyquist plots for G7Li at 25–300 °C temperatures. The equivalent circuit for fitting the ac-impedance data is also shown.

The ionic conductivity of G7Li is estimated by ac-impedance spectroscopy (c/f ESI). Figure 4 shows the Arrhenius plot of ionic conductivity versus temperature (relative humidity, RH = 39%). The impedance data typically in the low conductivity regime comprised of a single semicircle. This data could be approximately fitted by a resistance (*R1*) and CPE1 (=*R*^*n*−1^*C*^*n*^) in parallel^5^,^6^,^7^,^8^. In the high conductivity regime (i.e. (200–300) °C and around room temperature) the impedance data comprised of a depressed semicircle and a “spike-like” region at high and low frequency region respectively. The impedance data in the high conductivity regimes are fitted to a series combination in R1, 1 should be in normal mode and not suffix. It is written as R1 (*R*_*1*_) and constant phase element, CPE1 in parallel and CPE2 using ZView^TM^ software (Scribner Associates Inc.). The impedance data, in general could be fitted well with *n* = 0.8 resulting in bulk capacitance values ∼10^−11^ F. Assuming the “spike-like” region to be another depressed semicircle, reminiscent of ceramic conductors, fitting of the “spike-like” region is also attempted using a resistance (*R2*) in parallel to CPE2. In this case, the value of *n* which fitted the data is found to be low (=0.5) which resulted in capacitance values ∼10^−6^ F. As G7Li is not a ceramic conductor and hence does not possess well defined grain boundaries, it is strongly felt that this model here may not be appropriate. The impedance response of G7Li is very similar to a soft-matter electrolyte such as polymer electrolyte with the additional advantage of in-built lithium ions without the requirement of addition of any external salts. As the aim here is to solely estimate the bulk contribution, the ionic conductivities are estimated from resistance values as per the equivalent circuit depicted in Fig. 4. The room temperature conductivity is found to be ∼1.0 × 10^−7^ Ω^−1^ cm^−1^, which reduces to 3.9 × 10^−11^ Ω^−1^ cm^−1^ at 100 °C (Fig. 4). Interestingly, the conductivity increased sharply with further increase in temperature and reached a value of 0.2 × 10^−5^ Ω^−1^ cm^−1^ at 300 °C. Upon cooling, the conductivity retraces its path in both the temperature regimes i.e. 100–300 °C and 25–100 °C about the point of minimum (c/f Figure S6). The conductivity (σ) in the 100–300 °C temperature regime can be fitted to the Arrhenius equation, *σ* = *A*exp(−*E*_*a*_/*kT*) where *A* is the pre-exponential factor, *k* the Boltzmann constant, *T* the absolute temperature and *E*_*a*_ being the activation energy. The activation energy is estimated to be ~0.8 eV in the temperature range 100–300 °C (c/f Fig. 4).

The room temperature ionic conductivity is accounted by the water assisted lithium ion motion in G7Li. The observed decrease in ionic conductivity up to 100 °C can be attributed to the loss of water molecules, as evident from the TGA profile (c/f Fig. 3a). Following the evaporation of water molecules at *T* > 100 °C, the lithium ions primarily interact via guanine ligand. It is highly probable that lithium ion transport takes place through a hopping-like mechanism involving coordination to various sites in the guanine ligand leading to an Arrhenius-type behavior of conductivity versus temperature^44^. This is also supported by the estimated activation energy (=0.8 eV; T = (100–300) °C; anhydrous phase) which is similar to that of some of the known class of inorganic lithium ionic conductors in the same temperature range^45^ (as the conductivity decreases from room temperature to 100 °C, activation energy is not estimated as it will be unphysical). To further support the results of ionic conductivity of G7Li, differential scanning calorimetry (DSC) analysis is also performed (inset of Fig. 3a). To understand the non-monotonic behaviour of ionic conductivity of G7Li, DSC for two cycles of heating and cooling in the same temperature range (25–300 °C) as that of the conductivity experiments are performed. In the first heating scan, G7Li (inside a hermetically sealed DSC aluminium can) shows a broad and strong endotherm centering at 101.7 °C (∆H_transition_ = −167.9 J g^−1^), along with two more major endotherms at 162 °C (∆H_transition_ = −74.4 J g^−1^) and 281.6 °C (∆H_transition_ = −67.5 J g^−1^). While the endotherm at 101.7 °C corresponds to phase transition following the loss of water, the two other endotherms at 162 °C and 281.6 °C may correspond to the transitions from the rod-like morphology to the poorly ordered (irregular shape) phase of the compound which is clearly evident from SEM images (c/f Fig. 3b,c). This is additionally supported by the TGA profile which shows no appreciable loss (weight loss in the dehydrated phase in 80–300 °C: 0.8%) at these transition temperatures. The PXRD pattern of the sample pre-heated to 300 °C (c/f Figure S5) also broadened as compared to the as-synthesized sample. Upon cooling the sample in the reverse cycle (300 → 25 °C) no DSC exotherms are observed indicating that the initial (rod-like morphology) phases are not recovered. Following the 1^st^ heat and cool cycles, a small hole is made in the lid of the aluminium can and the sample is kept in air (≈ RH 39%) for two days. This sample is again taken for the 2^nd^ set of heating and cooling DSC experiments (c/f Figure S7). The regeneration of the endotherms in the second heating cycle and no appearance of exotherms again in the subsequent cooling cycle strongly suggest that the conductivity behaviour below the point of minimum (c/f Fig. 4) is water assisted lithium transport. Interestingly, when the 2^nd^ heating cycle from room temperature to 300 °C using the same heating rate is done under inert conditions (i.e. samples inside hermetically sealed DSC aluminium can similar to 1^st^ heat and cool cycle), no endothermic peaks are observed (inset: Fig. 3a) which are clearly visible in the first heating cycle and the second heating cycle of the air-exposed sample. Upon cooling, similar to the first cycle no exotherms are observed. The observations from DSC and TGA suggest that while heating G7Li dehydrates till 100 °C. This accounts well the decrease in ionic conductivity with temperature till 100 °C. On cooling in air, the sample rehydrates to the same limit at around 100 °C and this again leads to increase in ionic conductivity. This strongly supports our conductivity versus temperature results where the conductivity is observed to retract the same path on cooling. To elucidate the structure of the dehydrated phase of G7Li, the sample is heated to a temperature of 150 °C and immediately encapsulated inside wax. The PXRD pattern of the dehydrated phase encapsulated inside wax (Figure S8) is identical to that of the as-synthesized sample (Figure S8a,c). This suggests that elimination of water molecules coordinated to Li^+^ ions in G7Li does not lead to any recognizable change in it’s crystal structure. This is probably due to the fact that the structure of G7Li is stabilized by infinite guanine ribbons in which each guanine moiety holds six hydrogen bonds (DDA···AAD type, D = donor and A = acceptor) with two adjacent guanine moieties (Fig. 1) and the π-π interactions between the guanine ribbons (Figure S1b). Thus the ionic conductivity at *T* > 100 °C is due to the hopping transport taking place because of lithium coordination to the carboxylate and carbonyl oxygens of guanine moiety in G7Li.

The lithium ion transference number (*t*_+_) in G7Li is estimated using the dc-polarization technique^46^,^47^ with non-blocking electrodes in a cell configuration of the type Li(metal)|G7Li|Li(metal) (ESI). The observed value of *t*_+_ = 0.75 suggest that 25% of the total ionic conductivity is sustained by other species viz. proton in G7Li. It is noteworthy to mention here that this value of *t*_+_ is very high as compared to conventional polymer electrolytes (≈0.3)^48^ and also higher than some of the rare reports on polycarbonate electrolytes with high Li^+^ ion transference number^49^,^50^. To investigate this issue, preliminary ionic conductivity of G7Li is performed under various relative humidity (RH) conditions of 52%, 75% and 98% in the temperature range of (21–35) °C. The sample pellets are exposed for sufficient time so as to reach equilibrium before recording the temperature dependent impedance measurements. The results of the ionic conductivity measurements as a function of relative humidity are shown in Figure S9 (Figure S10 shows the stability of G7Li pellet under humid condition). The ionic conductivity at 25 °C at RH equal to 52%, 75% and 98% are respectively 1.5 × 10^−7^, 1.5 × 10^−5^, and 1.0 × 10^−4^ Ω^−1^ cm^−1^. Thus, the value of conductivity at 98% RH is nearly three orders higher than the room temperature conductivity measured at 39% (c/f Fig. 4). At a nominal temperature of 35 °C, the ionic conductivities at relative humidity 52%, 75% and 98% are observed to be 2.5 × 10^−7^, 1.7 × 10^−5^ and 3.0 × 10^−4^ Ω^−1^ cm^−1^ respectively. The activation energies at different humidity levels are estimated to be 0.8 eV (52%), 0.7 (75%) and 0.6 (98%). Thus, the ion transport under higher RH conditions appears to be more facile with reduced activation energy as compared to ion transport under dryer conditions (c/f Fig. 4). The higher ionic conductivity may be due to either of the two reasons. Firstly, as the sample absorbs additional water under higher RH conditions, these results in higher dissociation of lithium ions and substantial enhancement in the water assisted Li-ion transport. Secondly, under higher humid conditions the proton transport may also be enhanced. Both these factors are non-trivial to probe using the transference number method employed in this study. Since under humid conditions the lithium metal electrodes will be unstable due to vigorous reaction with water, the deconvolution of individual contributions cannot be achieved using this method.

**Figure 5:**
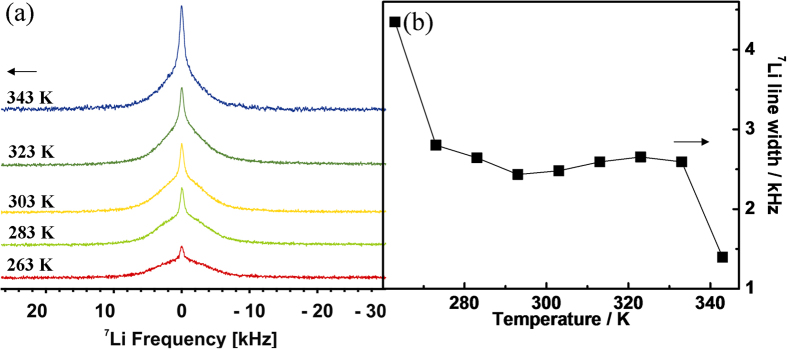
(**a**) Static ^7^Li-NMR spectra and (**b**) ^7^Li line-width (FWHM: full width at half maximum) as a function of temperature for G7Li solid powder.

The pulsed field gradient nuclear magnetic resonance (PFG-NMR) spectroscopy, an alternative tool to measure transference numbers of various ionic entities in a sample, also could not successfully deconvolute the individual diffusion coefficients for Li^+^ and H^+^ present in the sample due to their short T_2_ relaxation times as well as the slow diffusion. However, the Li^+^ ion mobility in G7Li is clearly recognized by monitoring the change in line-width (full width at half maximum, FWHM) as a function of temperature of the solid-state NMR spectra of ^7^Li in G7Li obtained from the stationary powder sample (c/f Fig. 5a). The ^7^Li being a quadrupolar nucleus (I = 3/2), the magnetic dipolar and electric quadrupolar interactions usually influence the ^7^Li NMR spectra (chemical shift interactions are normally small for ^7^Li). When a nucleus is fixed, the quadrupolar or internuclear dipole-dipole interactions are accentuated resulting in a broad line-width (usually known as rigid lattice line-width). The line-widths for G7Li (Fig. 5b) are obtained by fitting the spectra using a two components model consisting of the broad Gaussian peak (rigid lattice line-width) and the sharper Lorenzian peak corresponding to the central transition. The Li^+^ ion when it resides in a tetrahedral symmetry site, the quadrupolar contributions are expected to be negligible and so the ^7^Li line-width is in fact dominated by the dipolar interactions. In the range of temperature under investigation (−20 to 75 °C) no distinct quadrupolar satellite transitions (+3/2 ↔ +1/2 and −1/2 ↔ −3/2) are observed in the ^7^Li spectra of G7Li indicating that the Li^+^ ions are located at sites with tetrahedral symmetry (*T*_*d*_) and that there are no impurity ions^51^. This observation exactly supports the single crystal X-ray data. This concurrence also encouraged us to investigate the Li^+^ ion mobility by measuring the ^7^Li line-widths only of the central transition (+1/2 ↔ −1/2) as a function of temperature. At lower temperature (263 K) the spectrum is dominated by broad lattice line-width indicating that vast majority of the Li^+^ ions are relatively rigid, but still noticeable amount of mobile ones can be identified from the little narrow peak on top. The not so broad line-width of 4.3 kHz (Fig. 5b) of the central transition suggests that the Li^+^ ions may be mobile even at such a low temperature. As temperature increases, the line-widths gradually become narrower, (Figs 5 and S11). The line-widths steadily decrease to 2.4 kHz at 293 K (≈ambient temperature) after which it shows a slight increase to 2.7 kHz at 323 K. The steady decrease in line-width is due to higher mobility of Li^+^ ion at high temperature. Since broadening of line-width usually originates from the increased quadrupolar or internuclear dipole-dipole interactions^52^ the increase in ^7^Li line-width from 293 K to 323 K may be accounted on the basis of weak ^1^H-^7^Li dipolar interactions. This is also supported by the fact that the onset of motional narrowing of proton is also around 318 K (c/f Figure S11). This explains that the Li^+^ ion mobility below 373 K is water assisted as mentioned earlier and the decrease in ionic conductivity is also partially due to decrease in Li^+^ ion mobility in this temperature regime. The additional evidence of the water assisted movement of Li^+^ ion arises from the fact that Li^+^ ion hopping between the *T*_*d*_ sites, which are 6.991 Å and 4.261 Å (=*d*) apart (c/f Figures S2 and S4), is difficult. The jump period (*τ*) (ESI) at a particular temperature is related to the local diffusion coefficient (*D*) by the equation *D* = *d*^2^/4*τ*, and the obtained values are 1.22 × 10^−13^ and 0.74 × 10^−13^ m^2^/s for *d* = 4.261 and 6.991 Å respectively. Once water starts exiting from the system (*T* = 333 K) the ^1^H-^7^Li dipolar interactions weakens and this effect together with the increase in Li^+^ ion mobility reinitiate the ^7^Li motional line-width narrowing. The line-width data shows a sudden and sharp decrease between 333 K (2.6 kHz) to 343 K (1.4 kHz). The sudden increase in motional narrowing may be due to the loss of large number of water molecules during this stage. A further decrease of line-width is expected at and above 373 K, i.e. in the new dehydrated phase when water molecules have fully exited from the system. In the dehydrated phase within the temperature regime (373–573) K (c/f Fig. 4), there will also be an expected motional narrowing and it will purely be due to the fast Li^+^ hopping between the carboxylate and carbonyl oxygens of the guanine moieties in the G7Li. The activation energy calculated for the G7Li phase (≈21–40 °C) from the line-width measurements (c/f ESI) is 0.15 eV. Such low activation energies strongly support the water assisted diffusion of Li^+^ ions in G7Li at room and in proximity to the room temperature. Estimation of a lower activation energy barrier (compared to the ionic conductivity in the similar temperature range, Figure S9) can be anticipated primarily due to the differences in the probing methods and time scale of measurement between the ac-impedance and NMR techniques. The NMR detects specific ion motions on a much more local scale whereas the ionic conductivity data represents net number of mobile charges on a larger length scale^53^,^54^.

**Figure 6:**
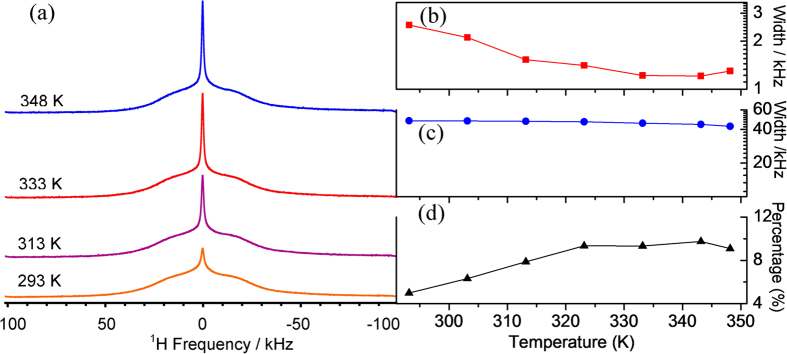
(**a**) Static ^1^H-NMR spectra, (**b**) ^1^H line width (FWHM: full width at half maximum) of the narrow component, (**c**) ^1^H line width of the broad component, and (**d**) the integration percentage of the narrow component as a function of temperature for G7Li solid powder.

To investigate whether protons in G7Li also contribute to the overall ionic conductivity as evident from the transference number measurements, the ^1^H solid state MAS NMR experiments are performed as a function of temperature. The ^1^H MAS NMR chemical shift spectra (c/f Figure S12a) shows two broad peaks centering at 1.04 ppm and 4.96 ppm corresponding probably to the free mobile water proton (Li → OH_2_) and the static proton covalently bonded to the nitrogen atoms of the guanine respectively. The broadening of the peaks partially might be a result of the hydrogen bonding in the system. The solid-state ^1^H MAS NMR spectra of G7Li (c/f Fig. 6a) are deconvoluted into a narrow component (mobile protons) and a broad component (rigid protons) using the mixed Gaussian and Lorentzian function (c/f example in Figure S12b) in order to obtain the line-width and integration for both the components. At 293 K the spectrum is dominated by the rigid lattice component (Fig. 6a), with recognizable contributions from the mobile protons. As the temperature increases the population of the mobile protons increases (Fig. 6d), suggesting the breach of the hydrogen bonding at elevated temperatures and thus more protons become mobile. This will negatively affect the conductivity as in ‘dry’ state the proton transport is achieved via breach and formation of hydrogen bonding (Grotthuss mechanism). The line-width that is observed to be 2.53 kHz at 293 K gradually decreases to 1.21 kHz at 343 K indicating an enhancement of proton mobility with temperature in the mobile region. However, an increase in line-width to 1.3 kHz with further increase in temperature to 348 K may be due to the onset of water molecules exiting from the system. The line-width of the rigid component is quite constant in the studied temperature range as shown in Fig. 6(c) indicating a quite stable structure and molecular dynamics in the lattice molecules. For proton to hop between different sites, the ideal distance between the sites normally is within 2.5 Å^55^,^56^. In G7Li the H···O distance between water molecules attached to two adjacent Li-tetrahedra (c/f Figure S4) is around 2 Å (<2.5 Å) suggesting that the facile proton conducting pathways is through these water molecules coordinately bonded to Li^+^ ions.

Despite the fact that G7Li shows Li^+^ and H^+^ ion conductivity under ambient conditions, it should however, be emphasized that the proton transport here is mainly due to the presence of water channels formed by water molecules tetrahedrally co-ordinated to the Li^+^ ions. This is further supported by NMR that out of the two types of protons (Figure S12a), the protons covalently bonded to the nitrogen atoms of a guanine moiety in an infinite guanine ribbons of G7Li (Fig. 1) are further held by the hydrogen bonds from neighboring two guanine moieties making them immobile. Further, as mentioned earlier, once the water is eliminated from G7Li by heating to above 100 °C it shows improved conductivity (even higher than the room temperature conductivity) purely due to the mobile Li^+^ ions. This also implies that eliminating water molecules under an inert condition will make the G7Li a purely Li^+^ ion conductor under ambient temperature. The enhanced Li^+^ ion conductivity at temperatures >100 °C is proposed here to be due to self-optimization of the ion hopping path lengths in a beneficial potential corridor formed by the carboxylate and carbonyl oxygens of the guanine moiety. The structural investigations of the single crystal of G7Li in the dehydrated phase using both single crystal X-ray diffraction and NMR are nearly impossible (as it quickly re-adsorbs atmospheric moisture to return to the room temperature phase) and this prohibits determination of the exact positions of Li^+^ ions in the dehydrated phase. However, as mentioned earlier powder X-ray diffraction studies by encapsulating the dehydrated sample in wax shows no appreciable change in crystal structure compared to the as-synthesized sample. This indicates that the positions of the Li^+^ ions are expected to remain the same both in the dehydrated phase and as-synthesized sample. This provides further proof of the fact that Li^+^ ions transport in the dehydrated phase of G7Li takes place through a beneficial potential corridor formed by the carboxylate and carbonyl oxygens of the guanine moieties in the infinite ribbons of guanine.

Cyclic voltammetry (CV) measurements at room temperature (=25 °C) are attempted to ascertain the stability window of G7Li. For the CV measurements (working electrode: stainless steel; counter and reference electrodes: lithium foil; scan rates: (0.25–1.0) mVs^−1^), a pellet of thickness 0.8 mm (diameter: 1.2 cm) measurements are taken. Thinner pellets could not be used as they tend to break and no attempt was made to cast thin films as they are beyond the scope of the paper. Under these measurement conditions, no electrochemical signatures including that of lithium deposition and stripping were observed due to high resistance of G7Li (not shown here). Further, as the conductivities are not so high at higher temperatures, performing the CV at elevated temperatures will also not be useful in precisely detecting the electrochemical stability. We do not discuss the electrochemical stability of G7Li any further in the absence of a scientifically sound CV data. In order to find out the Li-G7Li aerial specific resistance, the impedance spectrum of G7Li in a cell of the type Li|G7Li|Li is recorded. After fitting the corresponding Nyquist plot (Figure S13) with Maxwell equivalent circuit the Li-G7Li aerial specific resistance is found to be ~1.0 × 10^6^ Ω.

## Conclusions

In conclusion, we have demonstrated for the first time a novel bio-friendly solid electrolyte viz. G7Li obtained from a DNA base, guanine. G7Li, with it’s intrinsic supply of Li^+^-ions, exhibits a tunable room temperature ionic conductivity spanning over three orders in magnitude (10^−7^–0.3 × 10^−3^ Ω^−1^ cm^−1^) as a function of humidity. Two types of transport mediated by Li^+^ and H^+^ that contributes to the total conductivity in G7Li at ambient conditions are thoroughly deconvoluted using the transference number measurement and NMR line-width experiments. The transport above 100 °C is purely due to the Li^+^-ion. Considering the observations on temperature dependent ionic conductivity and structural analysis we strongly believe that G7Li may be beneficial as an electrolyte in conventional electrochemical devices and additionally for sensing applications especially humidity sensing. The novel approach demonstrated here may pave the way for the development of more biocompatible solid organic electrolytes and eventually lead to the replacement of liquid electrolytes that face severe problem of potential leakage of corrosive liquids and the volatility and flammability of the electrolyte solvents.

## Additional Information

**Data availability**: The CCDC contains the supplementary crystallographic data for this paper with the following deposition number of CCDC: 962004 (G7Li).

**How to cite this article**: Dutta, D. *et al.* N7-(carboxymethyl)guanine-Lithium Crystalline Complex: A Bioinspired Solid Electrolyte. *Sci. Rep.* **6**, 24499; doi: 10.1038/srep24499 (2016).

## Supplementary Material

Supplementary Information
