# Supplementary material for: N7-(carboxymethyl)guanine-Lithium Crystalline Complex: A Bioinspired Solid Electrolyte
Source: Sci Rep. 2016 Apr 19;6:24499. doi: 10.1038/srep24499 (PMC4835778; doi:10.1038/srep24499)
Supplement: Supplementary Information [file srep24499-s1.pdf]

Supplementary Information

N7-(carboxymethyl)guanine-Lithium Crystalline Complex: A Bioinspired Solid Electrolyte

Dipak Dutta,[‡,§] N. Nagapradeep,[‡,†] Haijin Zhu,[¶] Maria Forsyth,[¶] Sandeep Verma*[†,δ] and Aninda J. Bhattacharyya,*[§]

[§]Solid State and Structural Chemistry Unit, Indian Institute of Science, Bangalore-560012 (Karnataka), India

[†]Department of Chemistry, Indian Institute of Technology, Kanpur, Kanpur-208016 (UP), India

[δ]DST Thematic Unit of Excellence on Soft Nanofabrication, Indian Institute of Technology, Kanpur, Kanpur-208016 (UP), India

[¶]Institute for Frontier Materials, Deakin University, Waurn Ponds, VIC3216, Australia

EXPERIMENTAL

Materials and Methods

All solvents are distilled prior to use by using standard procedures. Solvents are evaporated using rotary evaporator under reduced pressure. All chemicals are of analytical grade.

Synthesis of N7-(carboxymethyl)guanine (1): N7-(carboxymethyl)guanine is synthesized by using our earlier reported protocol[1] which involve the synthesis of N^2-isobutyrylguanine and the intermediate (**I_1**) as depicted in Scheme-1.

Synthesis of N^2-isobutyrylguanine: N^2-isobutyrylguanine is synthesized according to the literature report of Jenny *et al*[2]. Mixture of guanine (3.54 g, 23.4 mmol) and isobutyric anhydride (10 g, 63.2 mmol) is heated for two hours at 150 °C in anhydrous DMF (50 mL). The reaction mixture is evaporated under reduced pressure and saturated aqueous $NaHCO_3$ solution added to it until effervescence ceases. The solution is filtered, the residue washed with water (2×20 mL) and dried under high vacuum (4.14 g, 80% yield). This crude product is used in the next step without further purification.

Synthesis of I_1: Intermediate I_1 is synthesized by the literature procedure of Liu *et al*[3]. To a suspension of N^2-isobutyrylguanine (4 g, 18 mmol) and NaH (0.6 g, 25 mmol) in anhydrous DMF (150 mL), ethylbromoacetate (2.22 mL, 19.9 mmol) is drop-wise added at 0 °C under N_2 atmosphere. The reaction mixture is stirred for two hours and then quenched with methanol. The reaction mixture is evaporated to a gummy solid and the required regioisomer I_1 is isolated via flash column chromatography eluting with 1% methanol/chloroform. (1.69 g, 50% yield, yellowish-white powder). HRMS: $(M+H)^+$ calculated: 308.1359, found: 308.1358; ^1H NMR (500 MHz, DMSO-d6, 25 °C, TMS): δ (ppm) 1.07 (d, 6H, two isobutyl CH_3's), 1.16 (t, 3H, ethyl CH_3), 2.70 (septet, 1H, isobutyl

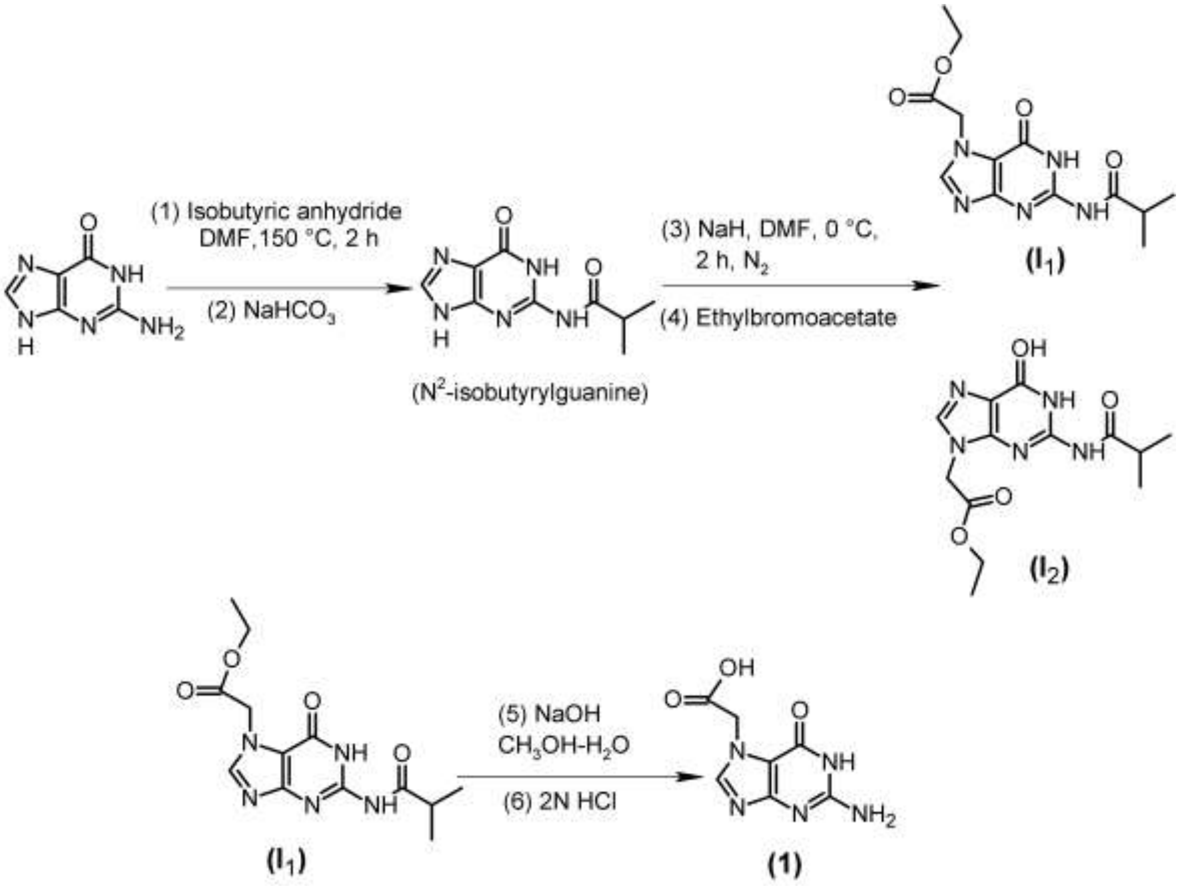

Scheme-1: Synthetic scheme for ligand **1.**

CH), 4.13 (q, 2H, ethyl CH_2), 5.16 (s, 2H, CH_2), 8.10 (s, 1H, C8-H), 11.55 (s, 1H, N1-H), 12.11 (s, 1H, N^2-H); ^{13}C NMR (125 MHz, DMSO-d6, 25 °C, TMS): δ (ppm) 14.50, 19.39, 35.24, 47.85, 61.87, 112.22, 145.44, 147.77, 153.17, 157.38, 168.38, 180.50.

Synthesis of N7-(carboxymethyl)guanine (1): Compound $\mathbf{I_1}$ (0.250 g) is dissolved in methanol (20 mL) and 2N aqueous NaOH solution added to it followed by stirring the reaction mixture for two days at ambient temperature. The required compound **1** is then precipitated out as a white solid by neutralization of the reaction mixture with 2N aqueous HCl solution. It is collected via filtration and finally dried under vacuum (0.125 g, 73%

yield). HRMS: $(M\text{-}H)^-$ calculated: 208.0476, found: 208.0473; ^1H NMR (500 MHz, DMSO-d_6, 25 °C, TMS): δ (ppm) 4.83 (s, 2H, CH_2), 6.18 (s, 2H, NH_2), 7.77 (s, 1H, C8-H), 10.89 (s, 1H, N1-H); ^{13}C NMR (125 MHz, DMSO-d_6, 25 °C, TMS): δ (ppm) 47.81, 109.03, 144.28, 153.32, 155.17, 159.96, 170.05.

Synthesis of G7Li: N7-(carboxymethyl)guanine (1) (0.020 g, 1 eq.) and $LiOH{\cdot}H_2O$ (0.016 g, 4 eq.) are dissolved in methanol/water mixture (4 mL, 1:4). The clear solution is kept undisturbed for a month and G7Li crystals are obtained on slow evaporation. These crystals are isolated and dried under high vacuum (0.006 g, 23% yield).

HRMS (positive mode): $[L+Li]^+$ calculated: 216.0709, found: 216.0720; $[2L+Li]^+$ calculated: 425.1258, found: 425.1306; $[(L-H)+L+2Li]^+$ calculated: 431.1345, found: 431.1135, $[2(L-H)+3Li]^+$ calculated: 437.1432, found: 437.1411, $[3L+Li]^+$ calculated: 634.1807, found: 634.1673, $[2L+(L-H)+2Li]^+$ calculated: 640.1894, found: 640.2137.

HRMS (negative mode): $[(L-2H)+Li]^-$ calculated: 214.0563, found: 214.0545; $[2(L-H)+Li]^-$ calculated: 423.1112, found: 423.1077; $[2(L-H)+2Li-H]^-$ calculated: 429.1194, found: 429.1154; $[2(L-H)+L+Li]^-$ calculated: 632.1661, found: 632.1650; $[3(L-H)+2Li]^-$ calculated: 638.1748, found: 638.1666, where L = N7-(carboxymethyl)guanine (1).

Crystal structure refinement details for G7Li: Single crystal of G7Li is coated with light hydrocarbon oil and mounted in the 100 K dinitrogen stream of a Bruker SMART APEX CCD diffractometer equipped with CRYO Industries low-temperature apparatus and intensity data are collected using graphite-monochromated Mo Kα radiation. The data integration and reduction are processed with the SAINT software[4] An absorption correction is applied.[5] Structures are solved by the direct method using SHELXS-97 and

refined on *F*2 by a full-matrix least-squares technique using the SHELXL-97 program package.[6] Non-hydrogen atoms are refined anisotropically. In the refinement, hydrogens are treated as riding atoms using SHELXL default parameters. Crystal structure refinement parameters are given in Table S1 whereas H-bonding parameters are provided in Table S2. CCDC contains the supplementary crystallographic data for this paper with a deposition number of CCDC **962004**. Copies of this information can be obtained free of charge on application to CCDC, 12 Union Road, Cambridge CB21EZ, UK. [Fax: +44-1223/336-033; E-mail: deposit@ccdc.cam.ac.uk].

Characterization:

^{1}H and ^{13}C NMR spectra are recorded on a JEOL-DELTA2 500 model spectrometer operating at ^{1}H Larmor frequency of 500 MHz. The spectra are recorded in DMSO–d_6 solutions and the chemical shifts are referenced with respect to tetramethylsilane. High resolution (ESI$^+$ and ESI$^-$ modes) mass spectra are obtained on WATERS HAB 213 machine. For thin layer chromatography (TLC), Merck pre-coated TLC plates (Merck 60 F_{254}) are used and compounds visualized under UV light at 254 nm. Flash chromatographic separations are performed on Merck 230-400 mesh silica gel.

The thermogravimetric analyses are performed by using the Perkin-Elmer Pyris 6 with a heating rate of 5 °C/min under N_2 atmosphere. Differential scanning calorimetry (DSC) is performed on a Mettler–Toledo model DSC 822$^©$differential scanning calorimeter, where temperature and enthalpy are calibrated with In (430 K, 3.3 J/mol) and Zn (692.7 K, 12 J/mol) standard samples using sealed Al sample pans. Cooling and heating profiles are recorded and analyzed using the Mettler–Toledo STAR$^©$software system. Data are collected from 25 to 300 °C with a heating rate of 5 °C/min.

Powder X-ray diffraction (PXRD) patterns are recorded on a PANalytical X-Pert PRO diffractometer with Cu-Kα radiation (1.5405 Å). Field emission scanning electron microscopy (FE-SEM) images are acquired on a FEI QUANTA 200 microscope, equipped with a tungsten filament gun and operating at WD 10.6 mm and 20 kV. Impedance spectroscopy measurements for estimation of ionic conductivity are performed on a Novocontrol-Alpha Impedance Analyzer in the frequency range of 1.0 MHz-10 mHz. The temperature variable ionic conductivity measurements at ambient condition are performed using a Thermolyne 1500 bench-top muffle furnace coupled with the impedance analyser. To perform the variable temperature impedance measurements under humid condition the impedance analyzer is coupled with a Julabo cryostat (Julabo FP50-HL Circulator) instead of the muffle furnace.

^{7}Li and ^{1}H line-width NMR (single pulse excitation) experiments are performed on a Bruker Advance III 300 MHz wide bore NMR spectrometer (7.05 Tesla) with ^{7}Li and ^{1}H Larmor frequencies of 116.64 MHz and 300.13 MHz respectively and equipped with a 4 mm double resonance Magic Angle Spinning (MAS) probe head. For 90° pulse the pulse length is 2.5 μs. The recycle delays (t) for ^{1}H and ^{7}Li are 30 s and 10 s respectively so as to allow the system to recover to the equilibrium state. For the variable temperature experiments, the actual sample temperatures are calibrated with lead nitrate, using the method described in the literature[7].

1. Nagapradeep, N., Sharma, S. & Verma, S. Ion Channel-like Crystallographic Signatures in Modified Guanine–Potassium/Sodium Interactions. *Cryst. Growth Des.* **13**, 455 (2013).

2. Jenny, T. F., Schneider, K. C. & Benner, S. A. N^2-Isobutyryl-O^6-[2-(p-Nitrophenyl)Ethyl]Guanine: A New Building Block for the Efficient Synthesis of Carbocyclic Guanosine Analogs. *Nucleosides & Nucleotides* **11**, 1257 (1992).
3. Liu, Z.-C. *et al*. Synthesis of photolabile *o*-nitroveratryloxycarbonyl (NVOC) protected peptide nucleic acid monomers. *Tetrahedron* **61**, 7967 (2005).
4. *SAINT+*, 6.02 ed.; Bruker AXS, Madison, WI, 1999.
5. Sheldrick, G. M. *SADABS 2.0*; University of Göttingen: Göttingen, Germany, 2000.
6. Sheldrick, G. M. *SHELXL-97*: *Program for Crystal Structure Refinement*; University of Göttingen: Göttingen, Germany, 1997.
7. Bielecki, A. & Burum, D. P. Temperature Dependence of ^{207}Pb MAS Spectra of Solid Lead Nitrate. An Accurate, Sensitive Thermometer for Variable-Temperature MAS. *J. Magn. Reson. Ser. A* **116**, 215 (1995).

Ionic conductivity from ac-impedance spectroscopy

Ionic conductivity is obtained from ac-impedance spectroscopy by scanning the sample in the frequency range 0.01–10^6 Hz (signal amplitude: 0.05 V). The pellet (diameter = 10.1 mm; thickness = 1.2 mm) of G7Li powder is sandwiched between two stainless steel electrodes in a home-built cell at ambient condition (25 °C). In order to check the reproducibility of the data and the compound stability, data are collected for two set of heating-cooling cycle on the same pellet. The G7Li pellet for the conductivity measurement is made at ambient condition (temperature ~25 ^{o}C; pressure applied = 50 kg/m^2). For the improvement in the interfacial contacts between the surfaces of G7Li pellet and stainless steel (SS) electrodes both surfaces of the pellet are

coated with a thin layer of gold. The impedance data typically in the low conductivity regime comprised of a single semicircle. This data could be approximately fitted by a resistance (*R1*) and CPE1 (= $R^{n-1}C^{n}$) in parallel[4-7]. In the high conductivity regime (i.e. (200-300) ^{o}C and around room temperature) the impedance data comprised of a depressed semicircle and a “spike-like” at high and low frequency region respectively. The impedance data in the high conductivity regimes are fitted to a series combination of resistance (R_1) and constant phase element, CPE1 in parallel and CPE2 using ZView[TM] software (Scribner Associates Inc.).

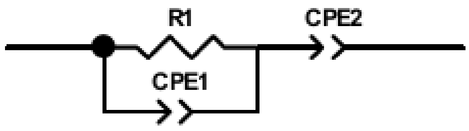

Scheme-S1. Equivalent circuit used for the analysis of the impedance plots for G7Li. Here, R1 is the bulk resistance and CPE1 and CPE2 are constant phase elements.

Fitting the data in the “spike-like” region by a resistance (*R2*) in parallel to CPE2 is also attempted similar to ceramic conductors[8-13]. In this case, the value of n which fitted the data reasonably is found to be low (= 0.5) which resulted in capacitance values ~ 10^{-6} F.

The transference number[14] measurements are done using a symmetrical two electrode cell as reported by Evans, J. *et al*[15,16]. A cell of the type Li(metallic)|G7Li|Li(metallic) is designed with a pellet of G7Li of diameter ~10 mm and thickness ~1 mm (G7Li pellet is sandwiched between two lithium electrodes). The lithium electrodes of similar dimensions as that of G7Li are obtained by punching lithium foil. The whole set up is then inserted into a cell as shown in Scheme-S2. The whole cell assembly is then sealed inside a pouch cell with only the connecting wires projecting outside. The sealing is done inside an argon filled glove-box

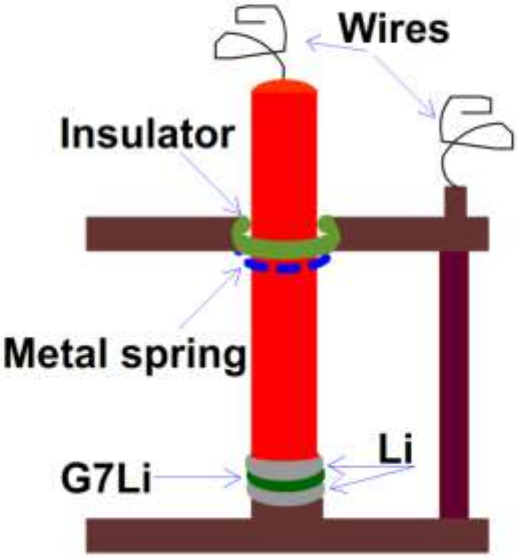

Scheme-S2. Schematic diagram for determination of transference number.

(H_2O < 0.5 ppm and O_2 < 0.5 ppm). The ionic conductivity prior and after current stabilization are evaluated using ac-impedance spectroscopy.[15]

8. Bayard, M. L.& Barna, G. G. A complex impedance plot of the ionic conductivity of $Na_{1+x}Zr_2Si_xP_{3-x}O_{12}$. *J. Electroanal. Chem.* **91**. 201 (1978).
9. Heitjans, P. & Indris, S. Diffusion and ionic conduction in nanocrystalline ceramics. *J. Phys.: Condens. Matter* **15,** R1257-R1289 (2003).
10. Croce, F., Appetecchi, G. B., Persi, L. & Scrosati, B. Nanocomposite polymer electrolytes for lithium batteries. *Nature* **394**, 456-458 (1998).
11. Appetecchi, G. B., Scaccia, S. & Passerini, S. Investigation of the stability of the lithium-polymer electrolyte interface. *J. Electrochem. Soc.*, **147**, 4448-4452 (2000).
12. Bronstein, L. M. *et al*. Design of organic-inorganic solid polymer electrolytes: synthesis, structure and properties. *J. Mater. Chem.* **14**, 1812-1820 (2004).
13. Pergolesi, D. *et al*. High proton conduction in grain-boundary-free yttrium-doped barium zirconate films grown by pulsed laser deposition. *Nat. Mater*. **9**, 846-852 (2010).
14. Spiro, M. in 'Techniques of chemistry', Vol. I, Part IIA, (Eds. A. Weissberger and B. W. Rossiter), Wiley, New York, 1970.
15. Evans, J., Vincent, C. A. & Bruce, P. G. Electrochemical measurement of transference numbers in polymer electrolytes. *Polymer* **28**, 2324 (1987).
16. Bruce, P. G., Evans, J. & Vincent, C. A. Conductivity and transference number measurements on polymer electrolytes. *Solid State lonics* **28-30**, 918-922 (1988).

Table-S1. Crystallographic data for G7Li.

**Identification code**	**G7Li**
Empirical formula	$C_{14}H_{24}Li_2N_{10}O_{12}$
*M*r	538.31
crystal system	Monoclinic
space group	$P2_1/c$
a/Å	14.889(5)
b/Å	9.842(3)
c/Å	7.840(3)
α/°	90
β/°	103.954(6)
γ/°	90
Volume/ $Å^3$	1115.0(6)
Z	2
Dx /Mg m^{-3}	1.603
F(000)	560
μ/ mm^{-1}	0.137
θ range for data collection/ °	2.82 to 28.29
Limiting indices	-19<=h<=19,<br>-13<=k<=12,<br>-9<=l<=10
Reflections collected	8568
unique reflections	2757
R(int)	0.0718
Completeness to θ	99.6
T_{max} / T_{min}	0.9730/0.9691
Data / restraints / parameters	2757/9/196
Goodness-of-fit on F^2	1.019
*R*1 and *R*2 [$I>2\sigma(I)$]	0.0579, 0.1349
*R*1 and *R*2 (all data)	0.0840, 0.1471
Largest diff. peak and hole/e.A^{-3}	0.443 and -0.328
CCDC No.	**962004**

Table-S2. Selected hydrogen bond distances (Å) and bond angles (°) in G7Li.

D—H…A#	D…A	H…A	D—H
**G7Li**			
N(1)—H(1)…N(3)^{i}	2.890(3)	2.01	177
O2W—H1W2…O(6)	2.865(2)	2.03(3)	171(3)
N(2)—H(2B)…N(9)^{i}	2.870(3)	1.99	175
N(2) —H(2A)...O(6)^{ii}	2.891(2)	2.06(3)	176(3)
O1W—H1W1...O(2)^{iii}	2.692(3)	1.85(2)	177(2)
O2W—H2W2... O(6)^{iv}	2.891(2)	2.06(3)	176(3)
O1W—H2W1...O(1)^{iv}	2.762(2)	1.96(2)	170(3)
O3W—H1W3...O(1)^{v}	2.872(3)	2.07(3)	165(4)
O3W—H2W3...O1W^{iv}	3.110(3)	2.53(3)	129(3)
O3W—H2W3...O2W^{iv}	3.114(3)	2.37(3)	152(3)

#Symmetry of A: (*i*)1-x,-1/2+y,1/2-z (*ii*) 1-x,1/2+y,1/2-z (*iii*) –x,-1/2+y,1/2-z (*iv*) x,1/2-y,1/2+z (*v*) -x,1-y,1-z where A = acceptor and D = donor.

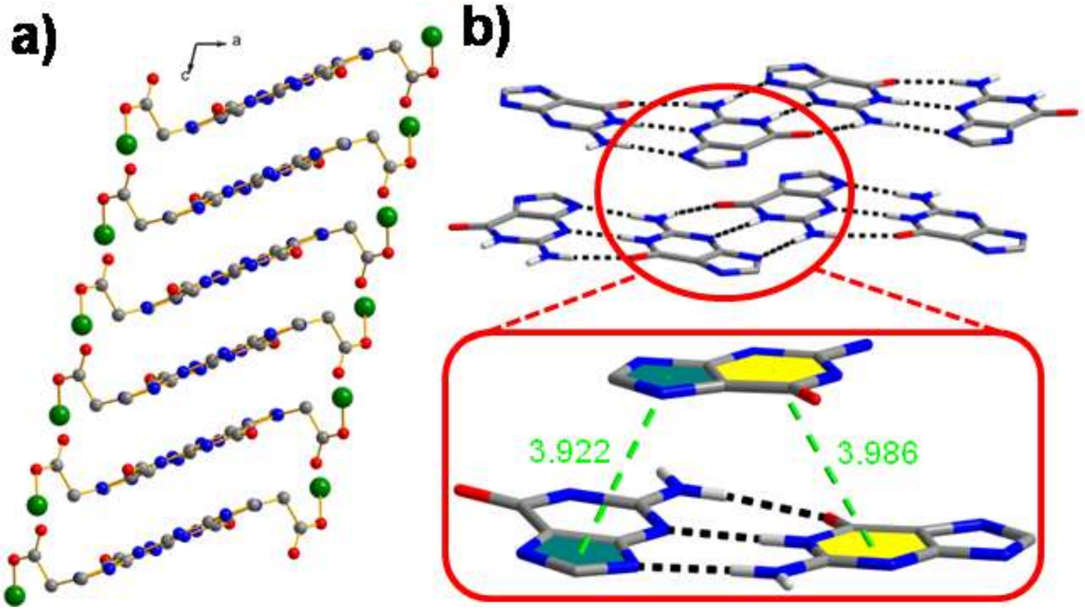

Figure-S1. a) Ladder–like lattice structure in G7Li when viewed along *b*-axis. (hydrogens and some of the water molecules are removed for clarity). b) π–π interactions (Å) in G7Li.

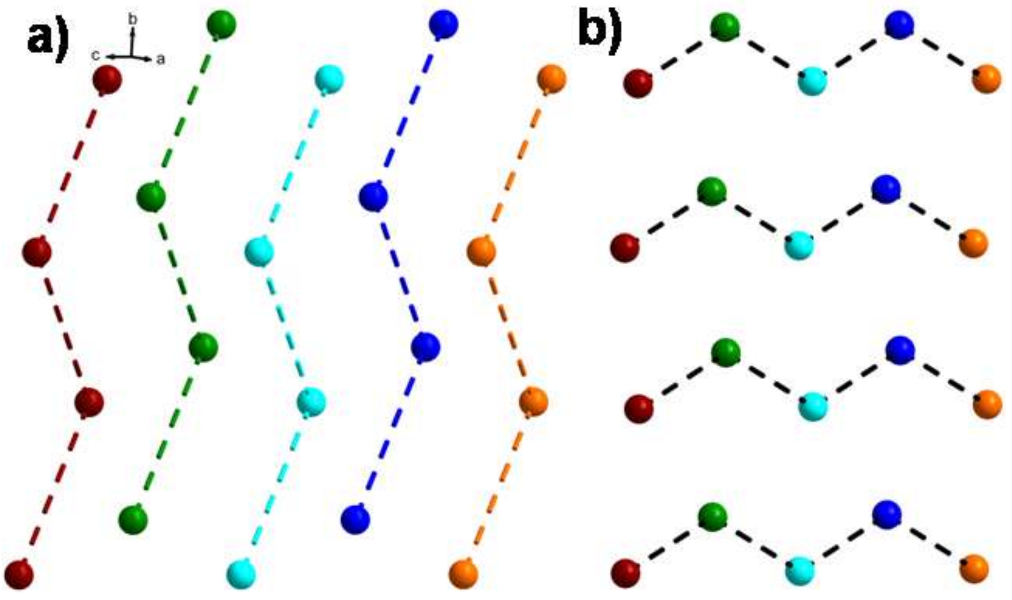

Figure-S2. Equidistant [dotted lines, (a) 6.991 Å and (b) 4.261 Å] alignment of Li ions (colored balls) in G7Li.

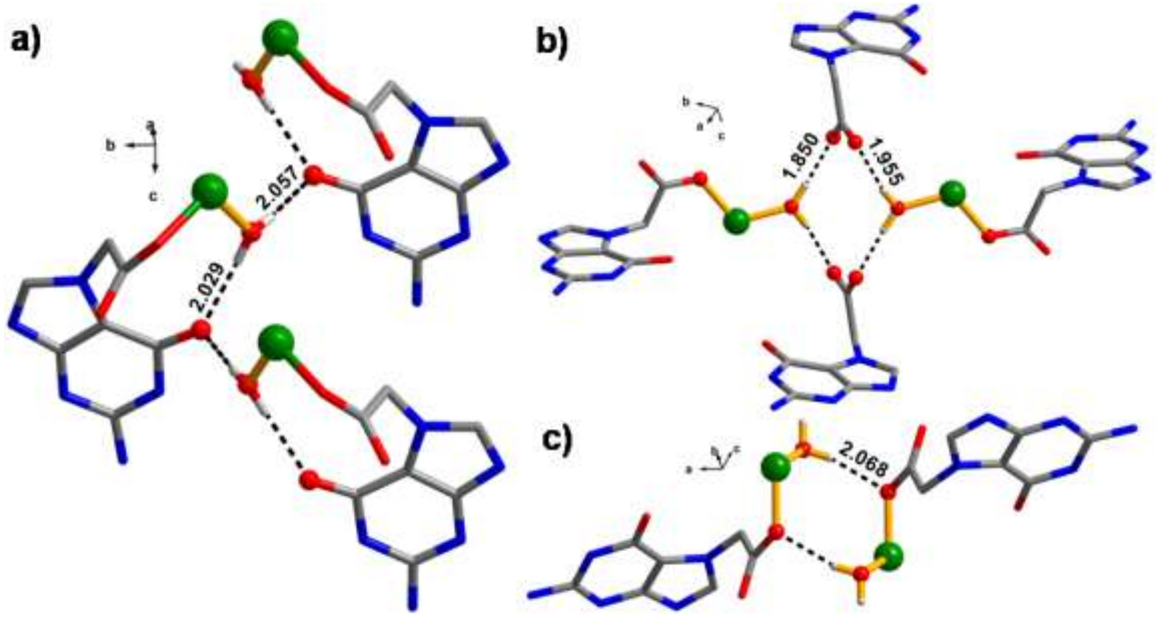

Figure-S3. (a) Hydrogen bonding interactions (Å) between Li–bound water molecules and O^6 of guanine in G7Li. (b) and (c) Hydrogen bonding interactions (Å) between Li–bound water molecules and O1 and O2 of guanine in G7Li.

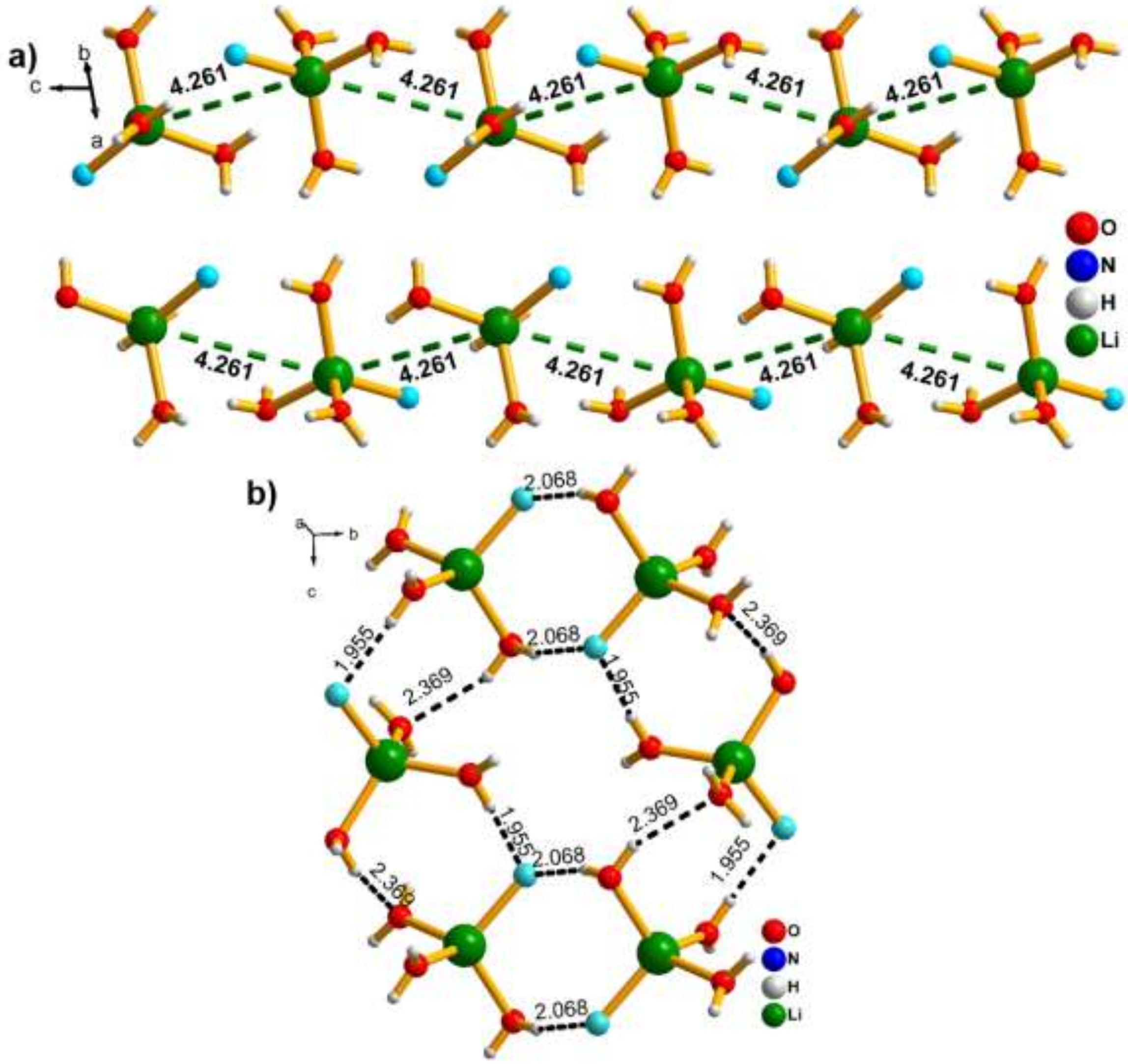

Figure-S4. Distances between (a) lithium and (b) proton conducting sites in G7Li. Color codes: skyblue balls → carboxylate oxygen attached to Li; red balls → water oxygens; light grey balls → hydrogen and dark green balls → lithium ions.

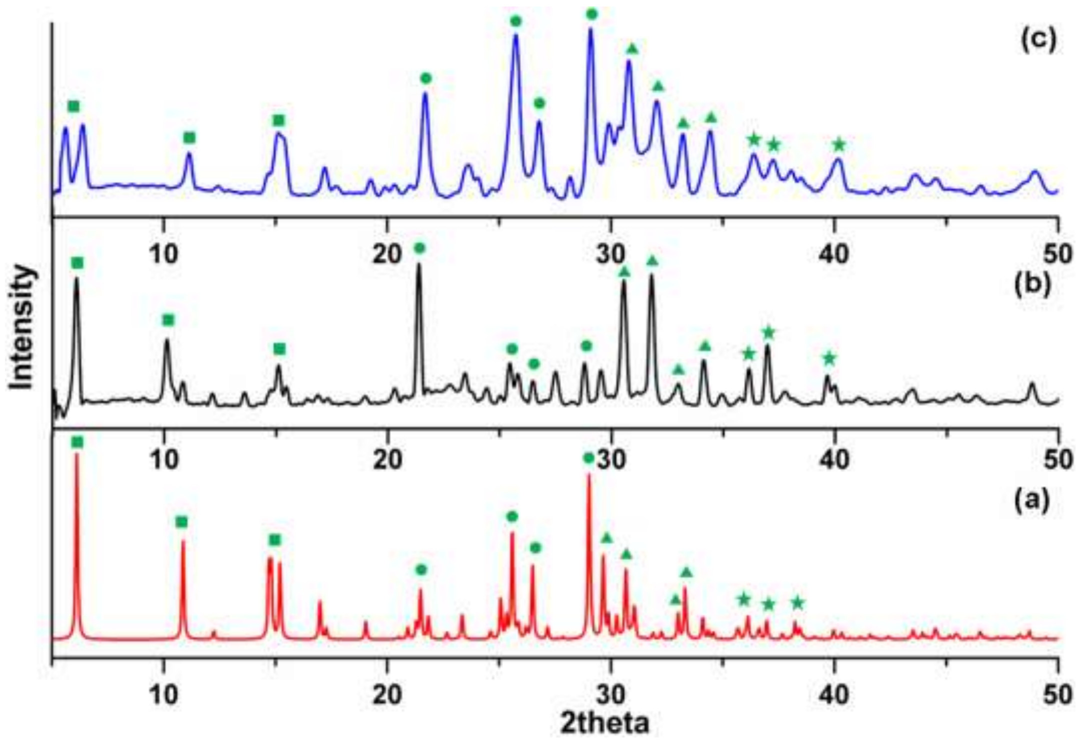

Figure-S5. PXRD patterns of G7Li. (a) simulated pattern at 100 K (from single-crystal X-ray diffraction), (b) observed pattern at 298 K and (c) XRD pattern observed after heating the sample at 573 K. The peak correspondence is marked with symbols. The shifting of some of the peaks may be attributed to the difference in temperature for simulated and observed PXRD data, which causes a difference in the inter-planar distances, thus changing the θ values.

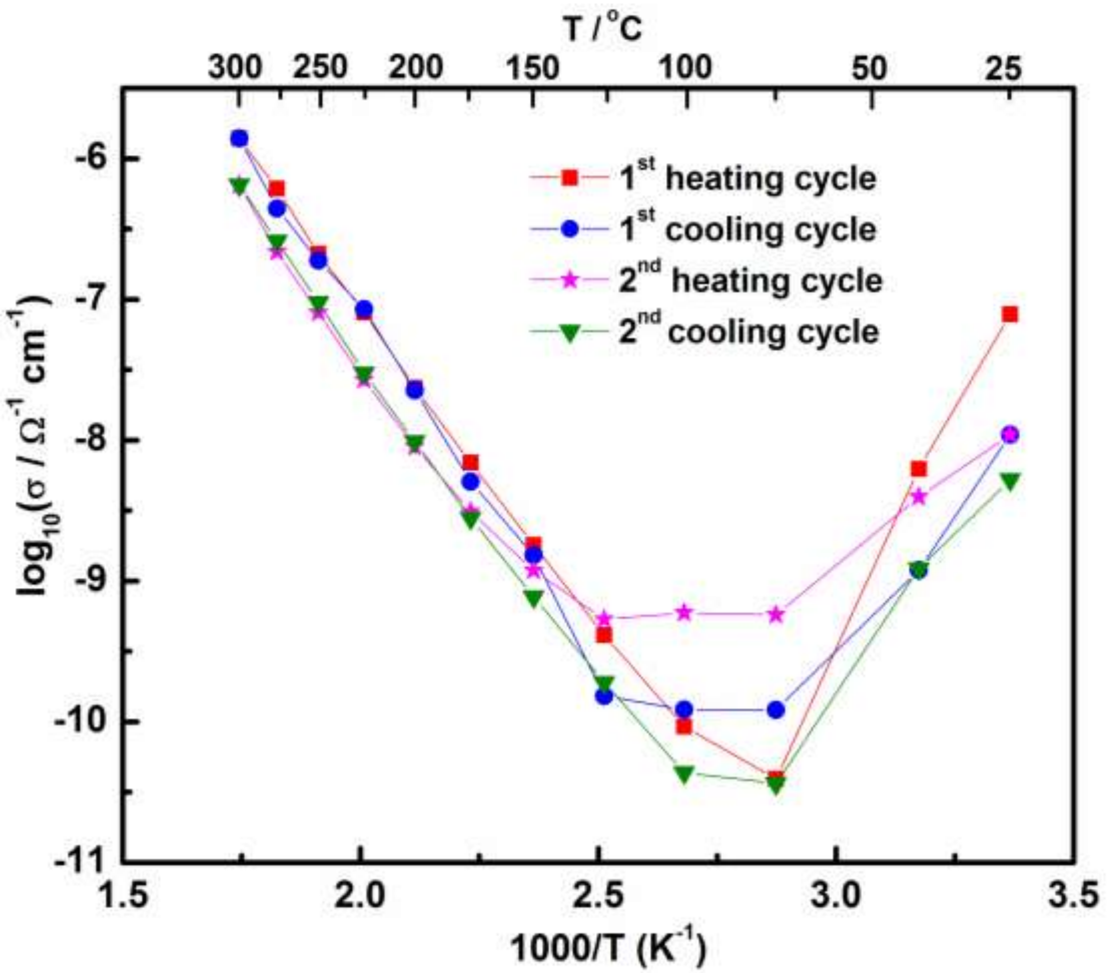

Figure-S6. Arrhenius plots of conductivity versus temperature (25-300 °C) for G7Li repeated over two heating and cooling cycles on the same pellet.

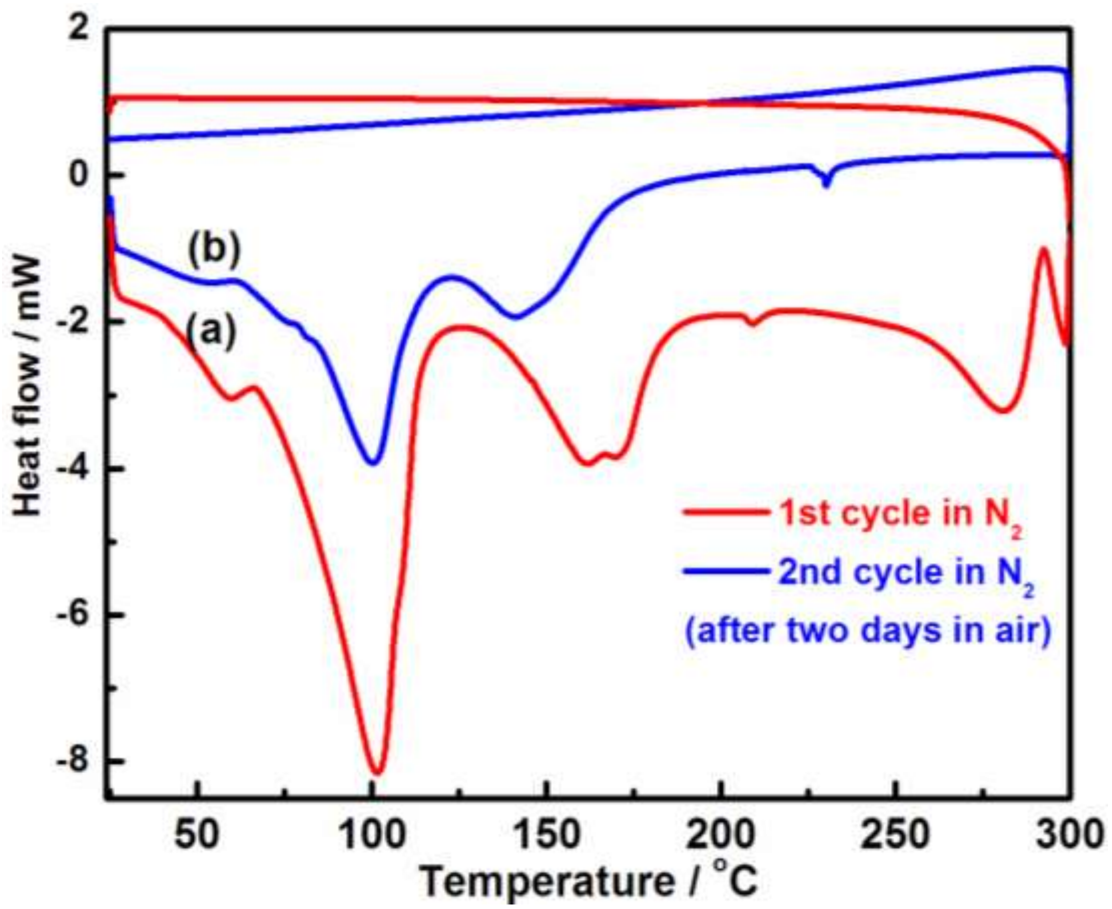

Figure-S7. Differential scanning calorimetry (DSC) profiles of G7Li: (a) first heating (25-300 ^{o}C) and cooling (300-25 ^{o}C) and (b) second heating (25-300 ^{o}C) and cooling (300-25 ^{o}C). The G7Li sample, after the first heating and cooling cycle had been over, is kept at ambient condition for two days so as to absorb sufficient moisture before performing the second set of heating and cooling.

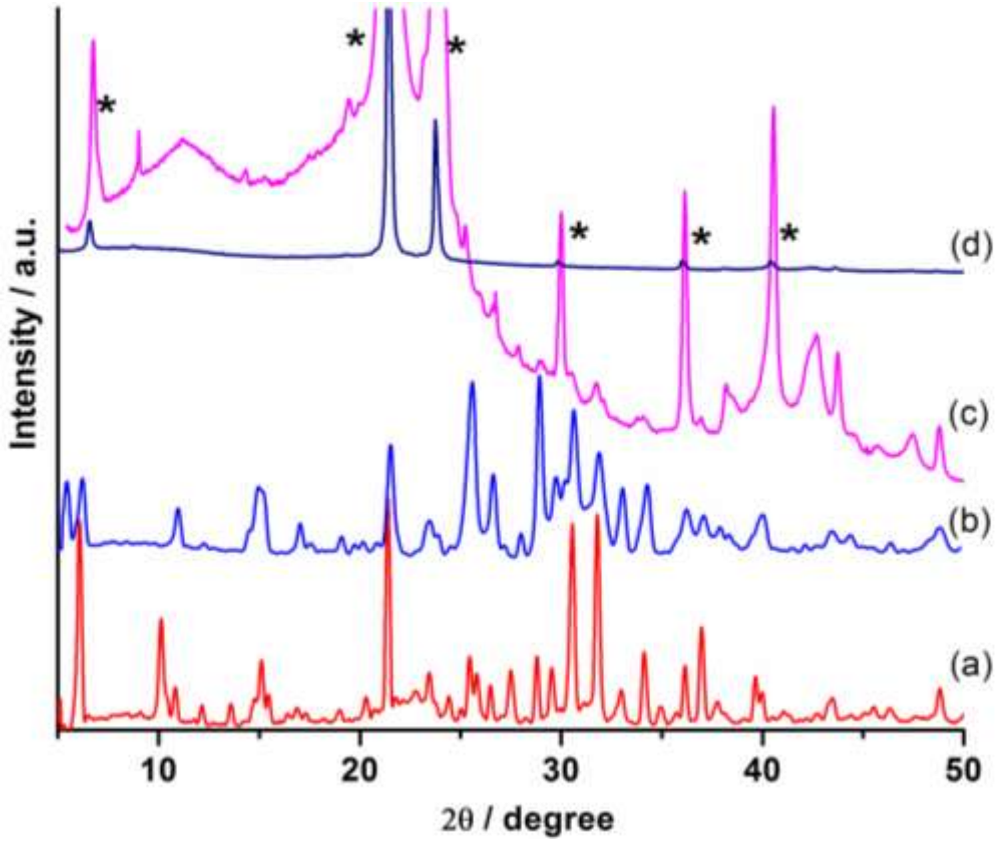

Figure-S8. PXRD patterns of (a) as-synthesized G7Li sample, (b) the sample pre-heated to 300 ^{o}C, (c) dehydrated phase of G7Li recorded by encapsulating the sample inside wax (the reflections indicated as ∗ are from the wax) and (d) the pristine wax.

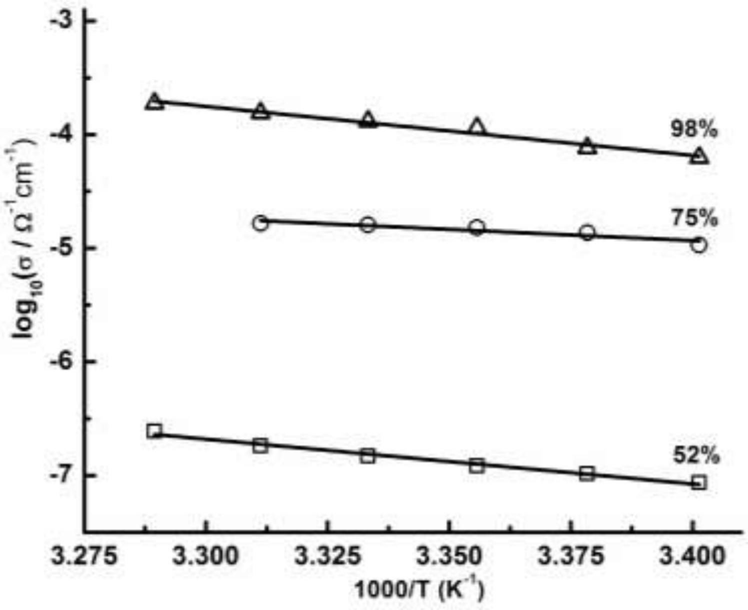

Figure-S9. Arrhenius plots of the conductivity versus temperature (21-31 ^{o}C) of G7Li at various RH conditions.

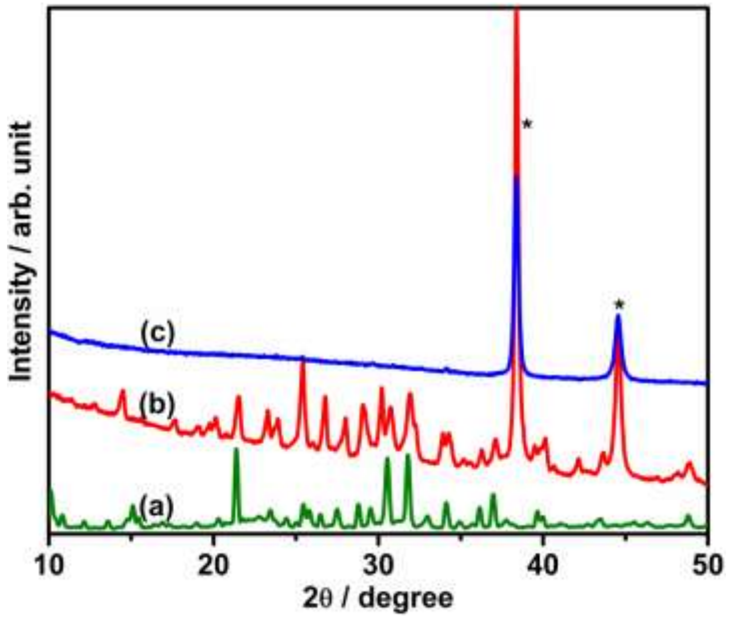

Figure-S10. PXRD patterns of G7Li (a) at ambient condition (298 K), (b) after exposure to 98% humidity, and (c) the silver paste used for coating both surfaces of the G7Li pellet. The extra peaks marked as * in XRD pattern of G7Li pellet (98% RH) is due to the silver paste.

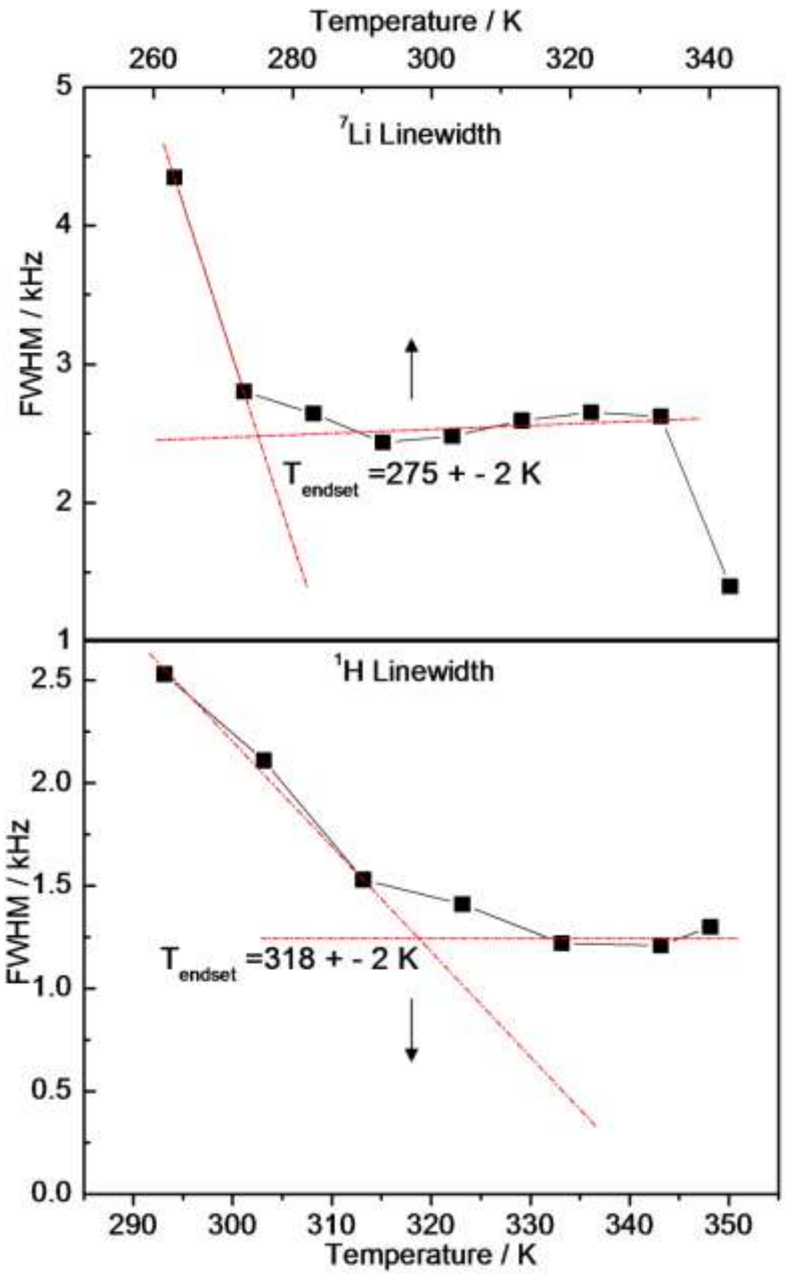

Figure-S11. Determination of the onset of narrowing temperature from the plot of line-width as a function of temperature for G7Li powder sample.

Determination of activation energy from the line-width measurement.

The activation energy of G7Li is obtained from the NMR line-width narrowing data as reported earlier.[17,18] It is known that the NMR motional narrowing of the ^{7}Li line-width takes place only when the rate of fluctuations of the local magnetic fields or electric field gradients (usually described by correlation time τ_c, also known as jump period (τ) is of the order of rigid lattice line-width (Δ_L), i.e.

$$1/\tau_c \approx \Delta_L$$

The correlation time is determined from the following equation

$$\tau_c = \frac{\alpha}{\Delta_T} \tan\left[\frac{\pi}{2} \left(\frac{\Delta_T}{\Delta_L} \right)^2 \right]$$

where Δ_T is FWHM at a given temperature and α a constant of the order of unity. Assuming t_c being thermally activated,

$$\frac{1}{t_c} = \frac{1}{t_0} \exp\left(-\frac{E_a}{kT} \right)$$

Linear fitting of $\log(1/\tau_c)$ vs. 1000/T in the range of temperature (21-40 ^{o}C), yields the activation energy.

17. Every, H. A., Zhou, F., Forsyth, M., MacFarlane, D. R. Lithium ion mobility in poly(vinyl alcohol) based polymer electrolytes as determined by ^{7}Li NMR spectroscopy. *Electrochimica Acta* **43**, 1465 (1998).

18. Saikia, D. *et al.* A new highly conductive organic-inorganic solid polymer electrolyte based on a di-ureasil matrix doped with lithium perchlorate. *J. Mater. Chem.* **21**, 10542 (2011).

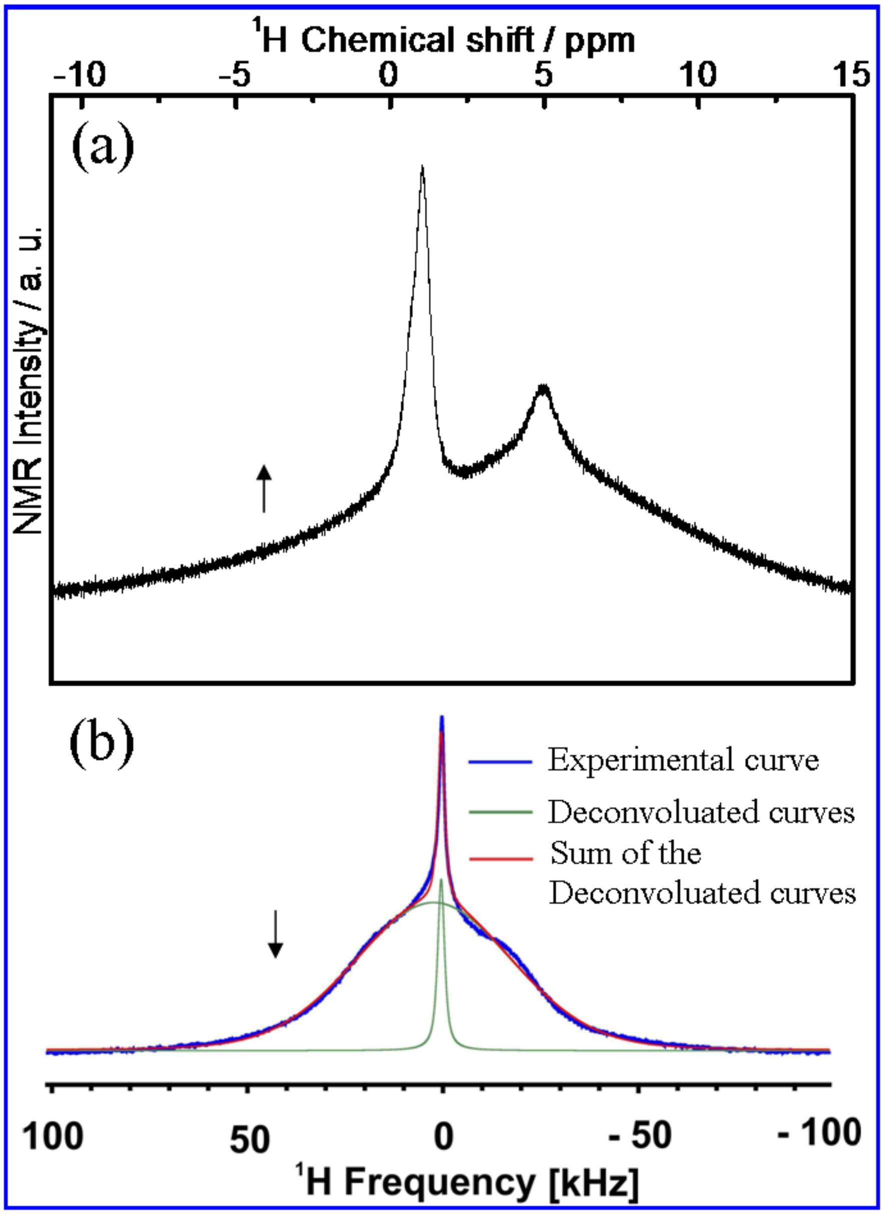

Figure-S12. (a) ^{1}H chemical shift and (b) an example of the curve deconvolution for the static ^{1}H NMR spectra of the G7Li powder sample.

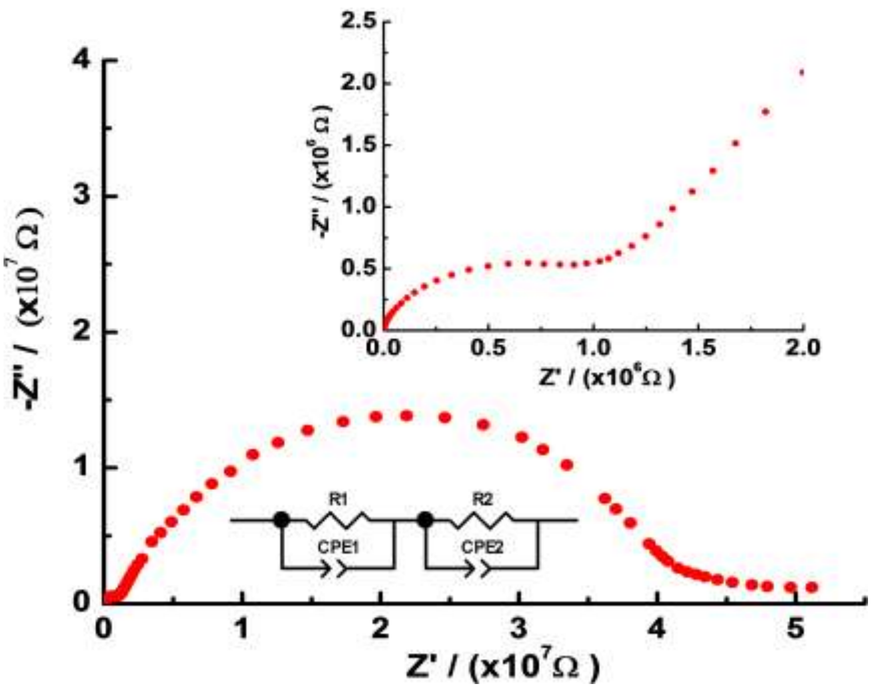

Figure-S13: (a) Nyquist plot of G7Li in a cell of the type Li|G7Li|Li showing both the high and low frequency semicircles. Inset: Nyquist plot showing only the high frequency semicircle. The equivalent circuit used for fitting the impedance data is also shown. Here CPEs are constant phase elements and R1 is the Li-G7Li aerial specific resistance.
